# Wounding causes rapid, intense upregulation of glycolytic and fermentative genes and enzymatic activities in harvested sugarbeet roots

**DOI:** 10.1038/s41598-025-05186-8

**Published:** 2025-07-01

**Authors:** Karen K. Fugate, Mercedes Morin, John D. Eide, Abbas M. Lafta, Fernando L. Finger

**Affiliations:** 1 USDA-ARS, Edward T. Schafer Agricultural Research Center, NCSL, 1616 Albrecht Blvd. N, Fargo, 58102-2765 ND USA; 2 Department of Plant Sciences, North Dakota State University, Fargo, ND USA; 3 Department of Plant Pathology, North Dakota State University, Fargo, ND USA; 4 Departamento de Agronomia, Universidade Federal de Viҫosa, Viҫosa, MG Brazil

**Keywords:** *Beta vulgaris*, Fermentation, Glycolysis, Postharvest, Storage, TCA cycle, Physiology, Plant sciences

## Abstract

Sugarbeet roots are severely wounded during harvest, triggering wound-healing responses to seal off and defend damaged cells. Primary carbon metabolism is required to provide metabolic energy and substrates for wound healing processes, yet how wounding alters primary carbon metabolism is largely unstudied in sugarbeet or other plant species. Wound effects on primary carbon metabolism were determined in the 24 h following injury by evaluating changes in gene expression and enzymatic activities of primary carbon metabolic pathways. Wounding significantly altered expression of 43 primary carbon metabolic pathway genes including 3, 19, 3, 7, and 9 genes involved in sucrolysis, glycolysis, TCA cycle/organic acid metabolism, pentose phosphate pathway, and fermentation, respectively. Highly upregulated genes were involved in sucrolysis, glycolysis, and fermentation, although only enzymatic activities of glycolytic and fermentative enzymes were majorly increased. The results indicate that wounding rapidly upregulates glycolysis and fermentation, with minimal effect on sucrolysis, the TCA cycle and the pentose phosphate pathway. We propose that glycolysis has a dominant role in controlling and upregulating carbon metabolism to support wound healing in postharvest sugarbeet roots, independent of elevations in sucrose catabolism or the TCA cycle, and fermentation is intensely upregulated to maintain glycolytic flux due to the insufficient activity of the TCA cycle to metabolize glycolytic end-products.

## Introduction

Sugarbeet (*Beta vulgaris* L.) roots sustain significant wounds from harvest and storage operations which negatively impacts their storage. At harvest, mechanical defoliators separate sugarbeet foliage from the taproot using knives or hard rubber flails that remove leaves and petioles but inflict cuts, scrapes, and bruises to the top of the taproot. Harvesters unearth taproots using lifters that break the taproot free from the lateral roots and the taproot’s tail which anchor it in the soil, bounce the harvested roots over chains or rollers to shake off soil, and drop them into truck beds. After transport, roots are dumped from trucks, agitated while they are lifted by piler elevators, and dropped onto piles for long-term storage. Injuries to roots from these operations are numerous and compound with each mechanical operation, such that all roots sustain cuts and abrasions before storage^1^^,^^2^. A survey of root damage additionally found within storage piles that 90% of roots sustained bruises greater than 2 cm^2^ in size, approximately 75% had wounds greater than 2 cm in diameter due to breakage, and nearly 50% had cracks exceeding 2 cm in length^3^.

Root injuries increase the rate of sucrose loss during storage by as much as 5 to 9.5-fold, as reported in two separate studies^4,5^. The increase in storage loss in response to injury is predominantly due to increases in the incidence and severity of storage rots, root dehydration, and elevations in endogenous root metabolism^6–8^. The loss of periderm from cuts, abrasions, cracks, and breaks provides sites for pathogens to enter and infect roots, with both the incidence and severity of storage rots increasing in proportion to the extent of root injury^7–9^. Breaks in the periderm additionally allow roots to dehydrate which also elevates storage losses due to both respiration and disease^6,10,11^. To minimize pathogen infection and water loss, metabolism is highly upregulated in wounded tissues to synthesize the compounds used to seal wound sites, produce metabolites and defense proteins with antimicrobial properties, and generate the compounds and energy needed to support cell division and growth that is a part of the wound healing response^12,13^. The metabolic pathways that are upregulated in response to injury are diverse as well as extensive, with a recent study finding 21% of expressed genes in the sugarbeet taproot were altered in expression in the 24 h after injury^14^.

The massive increase in anabolic metabolism that occurs in wounded roots necessitates an availability of carbon substrates and energy and requires alterations in primary carbon metabolism. Wounding increases sugarbeet root respiration by as much as three-fold^15,16^, and this respiratory increase likely occurs to provide energy to support the biosynthetic processes that are needed for wound-healing and root defense processes. In general, carbon substrates for secondary metabolic pathways are obtained by redirecting metabolic intermediates from primary carbon metabolic pathways. Substrates for cell wall biosynthesis are largely obtained from the cleavage of sucrose, catalyzed primarily by sucrose synthase^17^. Intermediates of the glycolytic pathway are redirected into metabolic pathways for the synthesis of lignin and suberin precursors, polyphenolic compounds, amino sugars, and lipids, or shunted into the pentose phosphate pathway for generation of substrates for nucleotide and aromatic amino acids, while tricarboxylic acid (TCA) cycle intermediates are diverted out of primary metabolism for the biosynthesis of purines, pyrimidines, fatty acids, aspartate, glutamate, and other amino acids^18,19^.

The mechanisms by which wounded sugarbeet root tissues increase the availability of carbon substrates to support upregulated biosynthetic processes is presently unknown. Elucidation of these mechanisms, however, is important for understanding postharvest wound-healing and defense responses, as well as carbon metabolism and sucrose loss during storage. Research was conducted to determine the effect of postharvest injury on the expression of genes involved in primary carbon metabolism in sugarbeet root. Initial research identified wound effects on the expression of genes that participate in sucrolytic, glycolytic and TCA cycle pathways since nearly all catabolism of sucrose in postharvest sugarbeet roots occurs via these pathways with little participation of the pentose phosphate pathway^20,21^. From this investigation, however, it was quickly apparent that genes involved in the pentose phosphate pathway and fermentation were also altered in expression in injured roots, so these genes were additionally included in this analysis. Enzymatic activities were quantified for genes, that based on their expression level and response to injury, were likely to have a role in the upregulation of carbon metabolism in wounded sugarbeet roots, since enzymatic responses to environmental stimuli often differ substantially from transcriptional changes^22,23^. Overall, the research presented here provides the first available information regarding the primary metabolic changes employed by sugarbeet roots to respond to the extensive injuries they sustain prior to storage.

## Results

### Carbohydrate metabolism

#### Sucrose catabolism

**Fig. 1:**
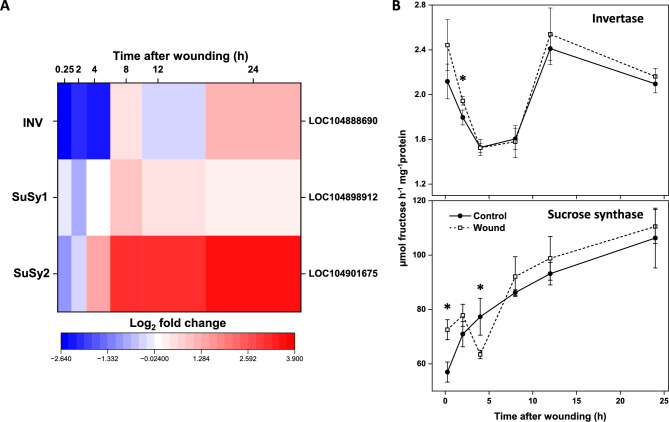
Effect of wounding on sucrolytic gene expression and enzyme activities during the first 24 h after injury. **A.** Heat map of the log_2_-fold change in expression of sucrolytic genes that were differentially expressed between wounded and control roots after 0.25, 2, 4, 8, 12, and 24 h after wounding. Only genes that were significantly different in expression between wounded and unwounded control roots at one or more time points are presented (p ≥ 0.05, FDR ≤ 0.01). Abbreviated gene names and gene identifiers are located on left and right axes, respectfully. Abbreviations: INV, soluble acid invertase; SuSy, sucrose synthase. **B.** Enzymatic activities of soluble acid invertase and sucrose synthase in wounded and unwounded control roots in the 24 h after injury. Error bars are equal to the standard error between replicates. Asterisks (*) denote time points at which enzyme activities were significantly different between wounded and unwounded control roots by t-tests (p ≤ 0.05).

Wounding of sugarbeet roots significantly altered expression of three genes involved in the catabolism of sucrose to its constituent monosaccharides: a soluble acid invertase gene (INV), catalyzing the hydrolysis of sucrose to fructose and glucose, and two sucrose synthase genes (SuSy1 and SuSy2) which catalyze the reaction of sucrose with UDP to produce fructose and UDP-glucose (Fig. 1A). Expression of the INV gene was downregulated in wounded roots in the first 4 h after injury, but was upregulated by 24 h to a level that was 2.2-fold greater than that of unwounded controls. Nevertheless, INV was expressed at extremely low levels at all time points regardless of wound treatment, with fragments per kilobase of transcript per million mapped reads (FPKM) values ranging from 0.1 to 6.3 (Supplementary Table S1). SuSy genes, in contrast, were highly expressed, with FPKM values of 457 to 1508 for SuSy1 and 198 to 2471 for SuSy2 (Supplementary Table S1). Both SuSy genes were upregulated in the 24 h following wounding, with SuSy1 and SuSy2 maximally upregulated by as much as 1.9-fold and 12.4-fold, respectively. Maximal expression of SuSy1 occurred at 8 h; maximal SuSy2 expression occurred after 24 h.

Despite the induction of sucrolytic gene transcription, root injury had minimal impact on sucrolytic enzyme activities (Fig. 1B). Wounding caused small, but statistically significant, differences in sucrolytic activity shortly after injury (2 h post-injury for INV activity; 0.25 and 4 h post-injury for SuSy activity). However, neither enzyme activity was affected by wounding between 8 to 24 h post-injury when gene expression was maximally upregulated. INV and SuSy activities, in contrast, were changed with respect to time after harvest, with INV activity transiently declining between 2 to 8 h and SuSy activity generally increasing in all roots regardless of wound treatment. Overall, SuSy activity far exceeded INV activity, with average SuSy activity 40-fold greater than average INV activity.

**Fig. 2:**
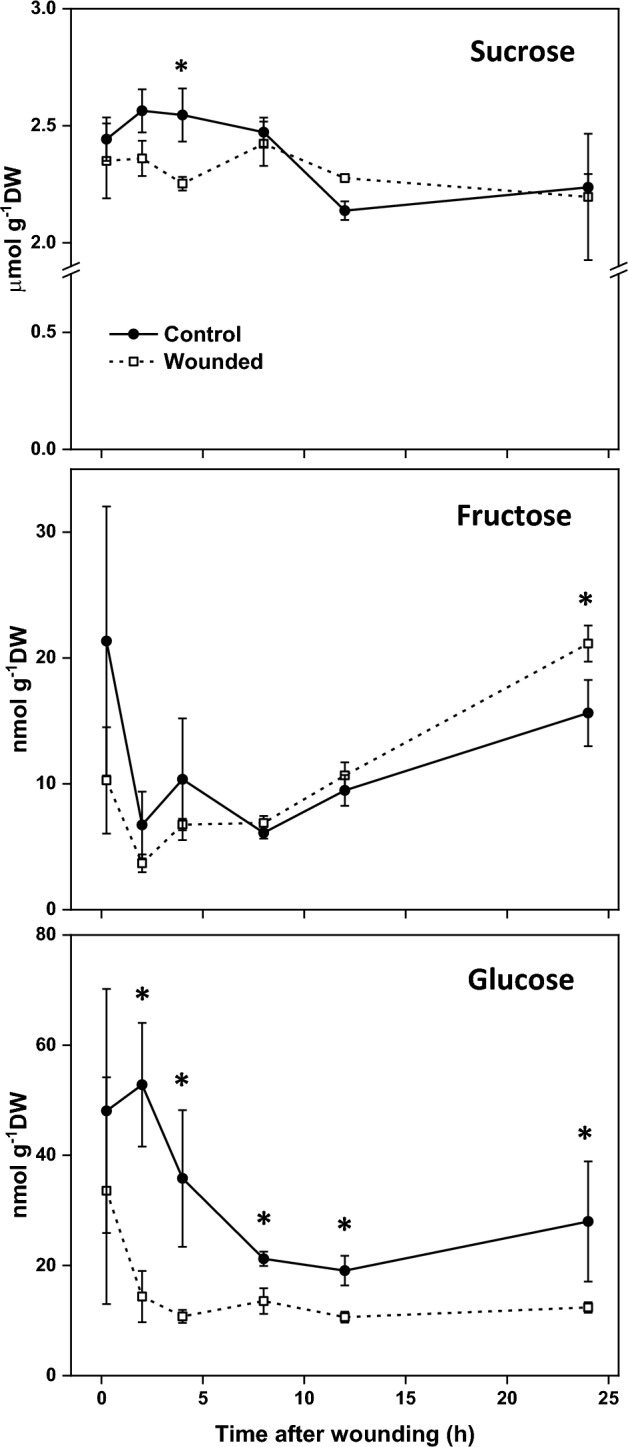
Changes in sucrose, fructose, and glucose concentrations in wounded and unwounded sugarbeet roots in the 24 h after injury. Error bars are equal to the standard error between replicates. Asterisks (*) denote time points at which concentrations were significantly different between wounded and unwounded roots (p ≤ 0.05).

Observed changes in SuSy and INV enzyme activities were generally reflected in the concentrations of sucrose and fructose, respectively (Fig. 2). Like SuSy activity, sucrose concentration in roots was generally unaffected by wound treatment but declined with time after treatment in inverse relationship to SuSy activity. Fructose concentration was also largely unaffected by wounding yet exhibited the same transient decline and recovery as observed for INV activity. Glucose concentrations in wounded and unwounded roots were unrelated to either sucrolytic enzyme activity. Unlike sucrose and fructose concentrations, glucose concentration was reduced by root injury throughout the 24 h following injury. Glucose concentration also declined sharply after harvest, regardless of wound treatment.

#### Glycolysis

**Fig. 3:**
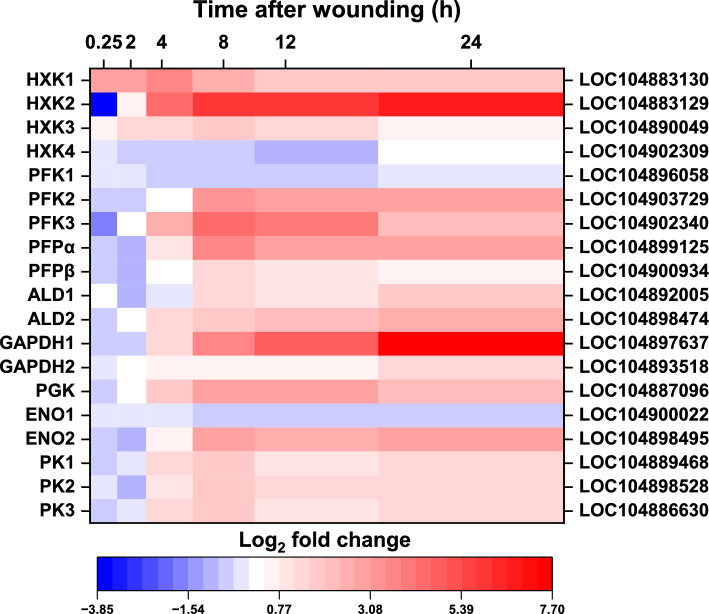
Effect of wounding on the expression of glycolytic pathway genes. Heat map of the log_2_-fold change in expression of glycolytic genes that were differentially expressed between wounded and control roots after 0.25, 2, 4, 8, 12, and 24 h after wounding. Only genes that were significantly different in expression between wounded and unwounded control roots at one or more time points are presented (p ≥ 0.05, FDR ≤ 0.01). Abbreviated gene names and gene identifiers are located on left and right axes, respectfully. Abbreviations: HXK, hexokinase; PFK, phosphofructokinase; PFP, pyrophosphate fructose 6-phosphate 1-phosphotransferase; ALD, aldolase; GAPDH, glyceraldehyde 3-phosphate dehydrogenase; PGK, phosphoglycerate kinase; ENO, enolase; PK, pyruvate kinase.

Nineteen genes encoding seven of the ten reactions that make up the glycolytic pathway were differentially expressed by wounding in the 24 h after injury (Fig. 3). These genes included four hexokinase (HXK) genes, three phosphofructokinase (PFK) genes, genes for the two subunits of pyrophosphate fructose 6-phosphate 1-phosphotransferase (PFP), two aldolase (ALD) genes, two glyceraldehyde 3-phosphate dehydrogenase (GAPDH) genes, a phosphoglycerate kinase (PGK) gene, two enolase (ENO) genes, and three pyruvate kinase (PK) genes. Fifteen of these genes (HXK1, HXK2, HXK3, PKF2, PFK3, PFPα, PFPβ, ALD1, ALD2, GAPDH1, GAPDH2, PGK, ENO2, PK2, and PK3) were upregulated by wounding, in most cases beginning within 4 h after injury. The most highly upregulated glycolytic genes in response to wounding were HXK2 and GAPDH1. In the 24 h following injury, HXK2 expression was 109-fold greater in wounded roots relative to unwounded control roots, while GAPDH1 expression was elevated 207-fold in wounded roots. Other genes that were both highly expressed and highly upregulated due to wounding included PFK2, PFK3, PFPα, and PGK which were maximally elevated ninefold, 20-fold, 12-fold, and eightfold, respectively, 8 h after injury, and ALD2 and ENO2 that were elevated sixfold and sevenfold, respectively, 24 h after injury. Pyruvate kinase genes, PK2 and PK3, were also highly expressed in harvested roots (Supplementary Table S1), but were more moderately upregulated by root injury with a maximum fourfold increase in expression. In contrast to the 15 glycolytic genes that were upregulated by injury, only four glycolytic genes (HXK4, PFK1, ENO1, and PK1) were downregulated by wounding. None of these genes, however, were highly expressed in wounded or control roots or downregulated by more than 55% relative to unwounded control roots (Supplementary Table S1). Additionally, a gene in the reverse, gluconeogenesis pathway, fructose 1,6-bisphosphatase, which catalyzes the dephosphorylation of fructose 1,6-bisphosphate to fructose 6-phosphate, was differentially expressed in wounded roots (Supplementary Table S1). Expression of this gene, however, was minimal in all roots and was not majorly affected by wounding.

**Fig. 4:**
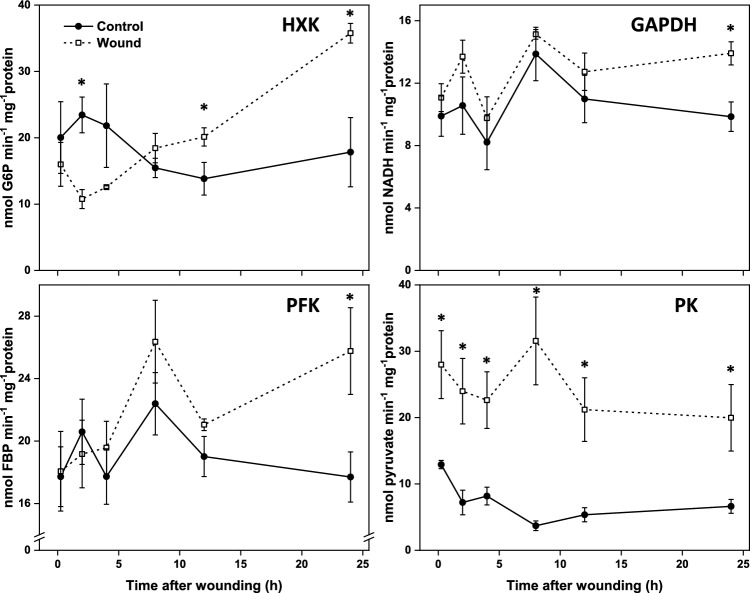
Enzymatic activity of hexokinase (HXK), phosphofructokinase (PFK), glyceraldehyde 3-phosphate dehydrogenase (GAPDH) and pyruvate kinase (PK) in wounded and unwounded control roots in the 24 h after injury. Error bars are equal to the standard error between replicates. Asterisks (*) denote time points at which enzyme activities were significantly different between wounded and unwounded control roots by t-tests (p ≤ 0.05).

The general upregulation in glycolytic gene expression in response to injury was reflected in increased enzymatic activities for HXK, PFK, GAPDH, and PK (Fig. 4). HXK, PFK, and GAPDH enzymes exhibited a general trend of increased activity over time in wounded roots, with activities that were significantly higher in wounded roots after 24 h compared to unwounded controls. PK activity was uniquely different from HKX, PFK, and GAPDH activities in both magnitude and duration of its response to wounding. PK activity was significantly higher in wounded roots throughout the 24 h after wounding and was elevated an average of 3.3-fold throughout the experiment (Fig. 4). The increase in PK activity was generally reflective of the increased gene expression of the PK2 and PK3 genes. These two PK genes are responsible for more than 96% of the PK transcripts in wounded or unwounded sugarbeet roots (Supplementary Table S1).

#### Pentose phosphate pathway

**Fig. 5:**
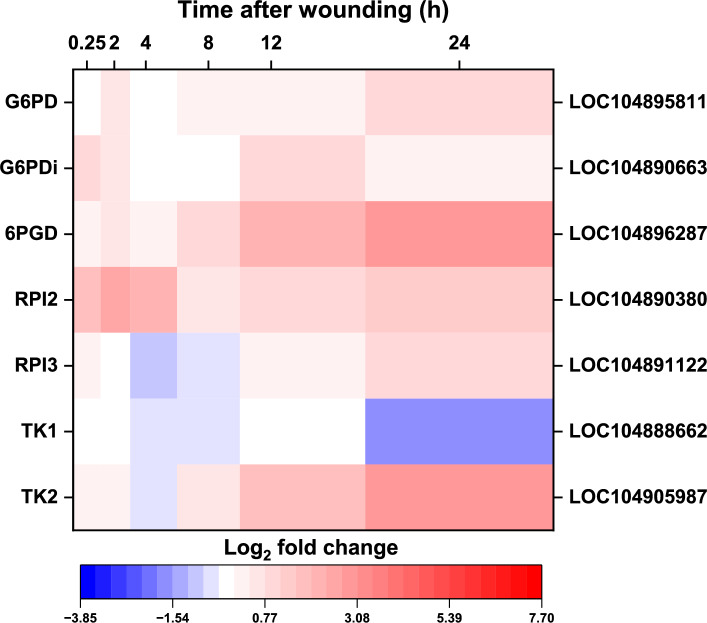
Effect of wounding on the expression of pentose phosphate pathway (PPP) genes. Heat map of the log_2_-fold change in expression of PPP genes that were differentially expressed between wounded and control roots after 0.25, 2, 4, 8, 12, and 24 h after wounding. Only genes that were significantly different in expression between wounded and unwounded control roots at one or more time points are presented (p ≥ 0.05, FDR ≤ 0.01). Abbreviated gene names and gene identifiers are located on left and right axes, respectfully. Abbreviations: G6PD, glucose 6-phosphate dehydrogenase; G6PDi, glucose 6-phosphate dehydrogenase, inactive; 6PGD, 6-phosphogluconate dehydrogenase; RPI, ribose 5-phosphate isomerase; TK, transketolase.

Wounding altered the expression of seven genes encoding four enzymatic activities of the pentose phosphate pathway (PPP). Six of these genes were upregulated by injury, including two glucose 6-phosphate dehydrogenase (G6PD) genes, a 6-phosphogluconate dehydrogenase (6PGD) gene, two ribose 5-phosphate isomerase (RPI) genes, and a transketolase (TK) gene, while a single gene, TK1, was downregulated by wounding (Fig. 5). A 6PGD gene and a TK gene (TK2) were elevated in expression by approximately eightfold at 24 h after wounding and were the most highly upregulated pathway genes, while the downregulated TK1 gene was maximally repressed threefold after 24 h (Supplementary Table S1). Although two genes encoding for G6PD, the enzyme generally considered to be rate-limiting for the pathway^18^, were upregulated by wounding, one gene (G6PDi), as per its GenBank annotation, encodes an inactive enzyme while the other (G6PD) was upregulated only after 24 h by a mere 2.2-fold increase in transcript levels relative to unwounded control roots (Supplementary Table S1).

#### Fermentation

**Fig. 6:**
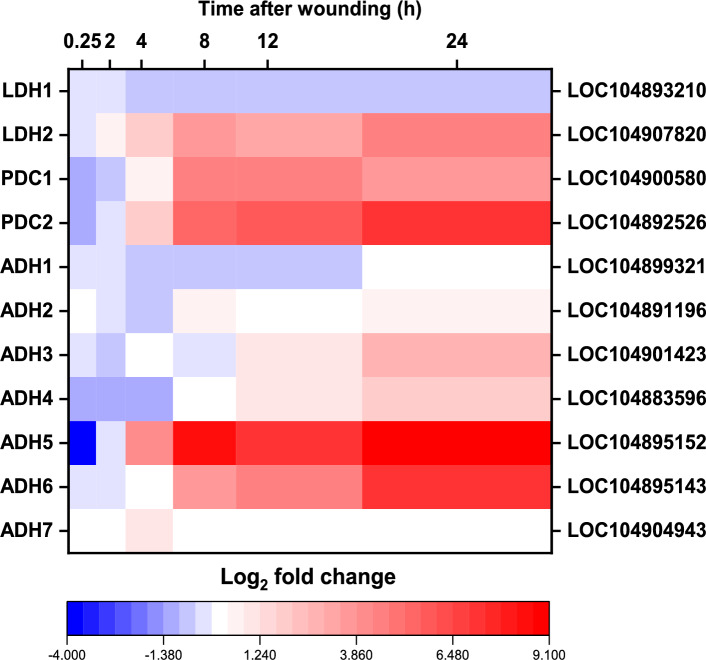
Effect of wounding on the expression of fermentative pathway genes. Heat map of the log_2_-fold change in expression of fermentative genes that were differentially expressed between wounded and control roots after 0.25, 2, 4, 8, 12, and 24 h after wounding. Only genes that were significantly different in expression between wounded and unwounded control roots at one or more time points are presented (p ≥ 0.05, FDR ≤ 0.01). Abbreviated gene names and gene identifiers are located on left and right axes, respectfully. Abbreviations: LDH, lactate dehydrogenase; PDC, pyruvate decarboxylase; ADH, alcohol dehydrogenase.

Wounding upregulated expression of nine genes involved in the fermentation of pyruvate to ethanol or lactic acid (Fig. 6). Two genes for pyruvate decarboxylase (PDC) and seven alcohol dehydrogenase (ADH) genes, which encode the two enzymes needed to convert pyruvate to ethanol, were highly upregulated in wounded roots in the 8 to 24 h following injury. PDC genes (PDC1 and PDC2) were highly expressed in wounded and control roots in the first two hours after harvest and initiation of treatments (Supplementary Table S1). While high PDC1 and PDC2 expression was maintained in wounded beets in the 24 h after injury, PDC1 and PDC2 expression in control roots declined as a function of time resulting in 22- and 125-fold greater expression of PDC1 and PDC2 genes, respectively, in wounded roots by 24 h (Fig. 6). Six of seven differentially expressed ADH genes (ADH2, ADH3, ADH4, ADH5, ADH6, and ADH7) were upregulated in wounded roots relative to controls, although greater than 96% of ADH transcripts in wounded roots were products of the ADH5 and ADH6 genes (Supplementary Table S1). These two genes were highly upregulated by wounding in the 24 h following injury, with their expression elevated by as much as 168- and 131-fold relative to unwounded roots. A lactate dehydrogenase gene, (LDH2), encoding the enzyme that converts pyruvate to lactate, was also elevated 25-fold in expression by wounding. LDH expression, however, was an average of 18- to 24-fold lower than that of the genes converting pyruvate to ethanol (Supplementary Table S1). Two genes encoding fermentative enzymes (ADH1 and LDH1) were downregulated by wounding. These genes, however, were minor contributors to ADH or LDH transcripts in roots regardless of wound treatment (Supplementary Table S1).

**Fig. 7:**
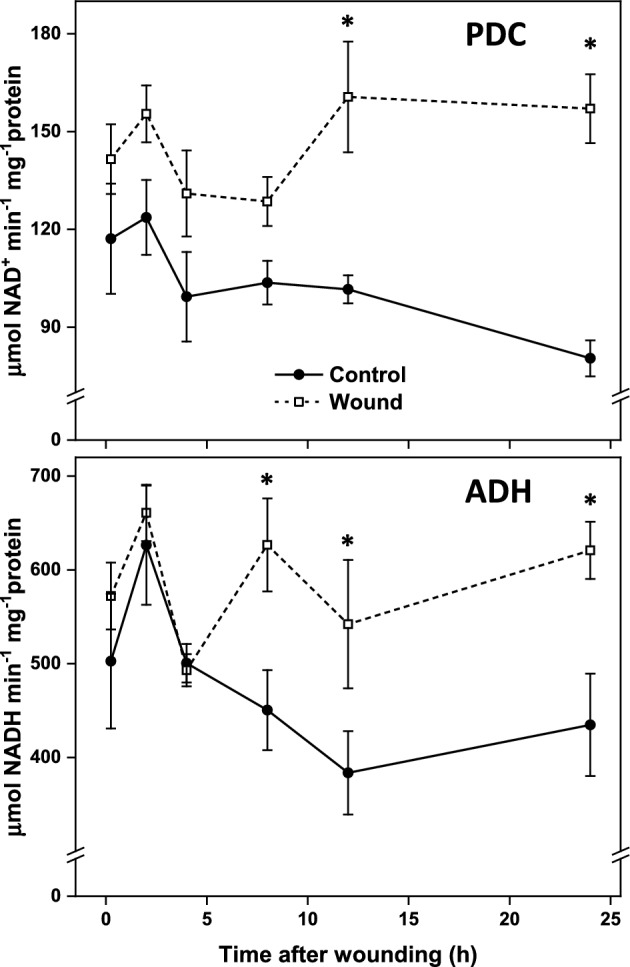
Enzymatic activity of pyruvate decarboxylase (PDC) and alcohol dehydrogenase (ADH) in wounded and unwounded control roots in the 24 h after injury. Error bars are equal to the standard error between replicates. Asterisks (*) denote time points at which enzyme activities were significantly different between wounded and unwounded control roots by t-tests (p ≤ 0.05).

Activities of the ethanol-producing fermentative enzymes, PDC and ADH, in wounded roots exceeded those of unwounded roots during the 24 h after injury (Fig. 7). Differences in PDC and ADH activities between wounded and unwounded roots, however, arose from relatively unaltered enzyme activities in wounded roots and declining activities in unwounded roots. PDC activity in wounded tissues was 1.7-fold higher than in unwounded roots in the 12 to 24 h after injury, and the ADH activity in wounded roots was an average of 1.85-fold higher in wounded roots in the 8 to 24 h after injury. The increased activity of these enzymes in wounded roots generally mirrored the elevations in expression of PDC1, PDC2, ADH5, and ADH6, which generated 96 to 100% of ADH and PDC transcripts, respectively, in wounded roots (Fig. 6, 7, Supplementary Table S1).

#### Tricarboxylic acid cycle and organic acid metabolism

**Fig. 8:**
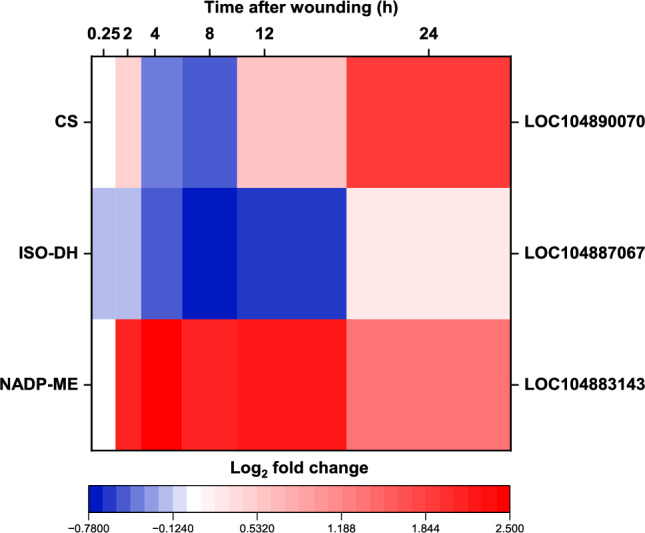
Effect of wounding on the expression of genes involved in tricarboxylic acid (TCA) cycle metabolism. Heat map of the log_2_-fold change in expression of genes involved in TCA cycle metabolism that were differentially expressed between wounded and control roots after 0.25, 2, 4, 8, 12, and 24 h after wounding. Only genes that were significantly different in expression between wounded and unwounded control roots at one or more time points are presented (p ≥ 0.05, FDR ≤ 0.01). Abbreviated gene names and gene identifiers are located on left and right axes, respectfully. Abbreviations: CS, citrate synthase; ISO-DH, isocitrate dehydrogenase; NADP-ME, NADP^+^-malic enzyme.

Wounding of sugarbeet roots had little effect on the expression of genes involved in the TCA cycle, with only two genes, citrate synthase (CS) and isocitrate dehydrogenase (ISO-DH), encoding enzymes catalyzing the first and third reactions of the TCA cycle, significantly altered in expression. Wound effects on the expression of these two genes were minor, with neither gene altered in expression by more than ± 1.9 log_2_-fold (Fig. 8). A NADP-dependent form of malic enzyme that decarboxylates malate, a TCA cycle intermediate, to produce pyruvate, the end-product of the glycolytic pathway, was also differentially expressed in wounded roots. This gene was maximally upregulated by 5.6-fold in the 4 h following injury.

## Discussion

An analysis of the effect of wounding on the primary carbon metabolism of harvested sugarbeet roots noted significant changes in the transcription of sucrolytic genes and genes of the glycolytic and the pentose phosphate pathways, the TCA cycle, and fermentation. The sheer number of pathway genes upregulated, the magnitude of transcriptional upregulation, and alterations in the enzymatic activities of upregulated genes, however, indicated that wounding greatly upregulated glycolytic and fermentation pathways with minimal effect on sucrolytic reactions, the pentose phosphate pathway, and the TCA cycle. These results suggest that the demand for carbon substrates and metabolic energy necessary to support sugarbeet root wound responses are largely met by elevating the capacities of glycolytic and fermentation pathways, with little or no activation of sucrolytic reactions or the TCA cycle.

### Glycolysis and fermentation are highly upregulated by wounding

#### Glycolysis

Fifteen genes encoding isozymes for seven of the ten reactions in glycolysis were upregulated following wounding. Genes encoding hexokinase, phosphofructokinase, pyrophosphate fructose 6-phosphate 1-phosphotransferase, aldolase, glyceraldehyde 3-phosphate dehydrogenase, phosphoglycerate kinase, enolase, and pyruvate kinase were upregulated by 4- to 207-fold in the 24 h after injury. Increases in transcription were reflected in increased enzyme activity for HXK, PFK, GAPDH, and PK, four enzymes implicated as possible regulators of carbon flux through the pathway^24–27^. The activities of three of these enzymes, HXK, PFK, and GAPDH, generally increased with time after injury and were significantly elevated 24 h following injury, while PK activity was elevated 2.1-fold within 15 min after wounding and remained elevated at levels ranging from 2.7-fold to as much as 8.8-fold compared to control roots in the remaining 24 h of the experiment.

The significant and sustained increase in PK activity due to injury was particularly notable since this enzyme is considered the primary controller of glycolysis for most plant species and organs^28,29^. PK carries out the final reaction in glycolysis and catalyzes the reaction of phospho*eno*lpyruvate with ADP to produce pyruvate and ATP. This enzyme has previously been implicated in the control of both glycolysis and respiration in postharvest sugarbeet roots^14,24^. An examination of changes in glycolytic enzyme activities and metabolites during storage identified PK as the primary regulator of glycolytic flux in postharvest sugarbeet roots^24^. In addition, PK activity and transcript abundance correlate with sugarbeet postharvest respiration rate, which is believed to be regulated by glycolytic substrate availability^24,30,31^. In the current study, two genes, PK2 (LOC104898528) and PK3 (LOC104886630), were highly expressed and upregulated by wounding at levels similar to the increase in PK activity in wounded roots. Whether one or both genes are responsible for the increase in PK enzyme activity, however, remains to be determined. Previously, PK2 and PK3 were reported to be highly expressed in postharvest sugarbeet roots throughout 120 d storage, with PK3 identified, via weighted gene co-expression network analysis (WGCNA), as a likely regulatory hub gene for storage root respiration rate^31^. While available evidence implicates PK as the major controller of glycolysis in postharvest sugarbeet roots, other glycolytic genes and enzyme activities upregulated by wounding, i.e., HXK, PFK, and GADPH, are also potential contributors to the control of glycolytic flux in wounded roots. These enzymes have previously been described as likely secondary regulators of glycolysis in sugarbeet as well as in other plant species^24,27,29,32,33^.

#### Fermentation

Wounding dramatically increased the expression of fermentative pathway genes and significantly increased the activities of the enzymes responsible for ethanol biosynthesis. Nine genes encoding all enzymes involved in the fermentation of pyruvate, the end-product of glycolysis, to ethanol or lactate were highly upregulated in the 24 after wounding. Genes for pyruvate decarboxylase and alcohol dehydrogenase, the two enzymes responsible for ethanol biosynthesis, were upregulated by as much as 125- and 343-fold, respectively, while a gene for lactate dehydrogenase which catalyzes the conversion of pyruvate to lactate, was upregulated by 25-fold. In sugarbeet, as in most plants, fermentation proceeds primarily toward the production of ethanol^34,35^. In contrast, fermentation to lactate generally serves to activate ethanolic fermentation, since acidification of the cytoplasm due to lactate production stimulates PDC enzymatic activity^36,37^. Enzymatic activities of PDC and ADH were elevated by as much as 2.0-fold for PDC, and 1.4-fold for ADH between 12 and 24 h after injury in wounded roots relative to controls. These elevated activities generally mirrored elevations in PDC1 and PDC2, and ADH5 and ADH6 gene expression, respectively, suggesting that PDC and ADH enzymatic activities were at least partially regulated by transcriptional changes.

The expression of fermentative genes and the activity of enzymes involved in fermentation pathways have not been previously reported in harvested or stored sugarbeet roots, at least to the authors’ knowledge. Fermentative pathways are most commonly induced in response to limited oxygen conditions in plants and have been shown to be activated in red beet root tissues incubated under anoxic conditions^34,38^. However, neither anaerobic nor reduced ventilation storage conditions were employed in the present study. Wound induction of fermentation has been reported in a single study which noted increased ADH enzymatic activity and the expression of new ADH isozymes in maize and lettuce seedlings following injury^39^. Induction of fermentation by drought or dehydration has also been documented, with elevations in PDC and ADH gene expression and ADH enzyme activity noted^40,41^. Since dehydration is a common consequence of wounding, upregulation of fermentation in response to dehydrating conditions may have relevance for the current study^6^.

### Sucrose cleavage, pentose phosphate pathway, and TCA cycle are minimally affected by wounding

#### Sucrose cleavage

Wounding upregulated the expression of genes involved in the cleavage of sucrose to its constituent monosaccharides but had little effect on sucrolytic enzyme activities. Two sucrose synthase genes, SBSS1 and SBSS2, are expressed in sugarbeet roots and are responsible for greater than 90% of the total sucrolytic activity in postharvest roots^42,43^. Wounding upregulated SBSS1 and SBSS2 expression by as much as 1.9- and 12.4-fold, respectively. An acid invertase gene was also upregulated 2.2-fold in the 24 h after injury, although expression of this gene remained at very low levels at all times during the experiment. Increases in sucrolytic transcripts in wounded roots, however, were not reflected in changes in sucrose synthase and acid invertase activities, suggesting that post-transcriptional mechanisms largely regulate sucrose synthase and acid invertase protein levels. Previous studies have similarly noted large discrepancies between gene expression and enzymatic activities of sucrose synthase and acid invertase in sugarbeet roots as well as other plant species and organs, validating the importance of posttranscriptional mechanisms in the regulation of these sucrose-degrading activities^22,44–46^. That wounding does not induce changes in sucrose catabolizing enzyme activities is supported by a previous study that found sucrolytic activities to be largely unaltered in wounded sugarbeet roots in the 13 d following injury^32^.

#### Pentose phosphate pathway

Although genes encoding four of the seven enzymatic activities that comprise the pentose phosphate pathway were upregulated by wounding, the upregulation of PPP genes was modest compared to the wound-induced upregulation of glycolytic and fermentation pathway genes. PPP genes were maximally upregulated 1.7- to 7.8-fold by wounding, in contrast to glycolytic and fermentation pathway genes that were upregulated by as much as 206-fold and 343-fold, respectively. Control of the flux through the PPP is largely attributed to glucose 6-phosphate dehydrogenase, the first committed enzyme in the pathway^47^. While a gene encoding for enzymatically active G6PD was upregulated by wounding, this gene was not significantly elevated until 24 h after injury when its expression was approximately doubled that of unwounded control roots. The pentose phosphate pathway was previously demonstrated to be an active, but minor contributor to overall carbon metabolism in harvested sugarbeet roots and operates to generate substrates for the biosynthesis of nucleotides, aromatic amino acids, and reducing equivalents in the form of NADPH^21,47^. The limited upregulation of this pathway in response to injury suggests that the PPP is active in wounded roots but likely remains a minor contributor to overall carbon metabolism.

#### TCA cycle

TCA cycle gene expression was largely unresponsive to wounding with genes for only two of the cycle’s eight enzyme activities differentially expressed in wounded roots. Genes encoding single isozymes of citrate synthase and isocitrate dehydrogenase were altered in expression due to wounding, with the ISO-DH gene largely down-regulated by injury, and the CS gene transiently downregulated between 4 to 8 h after injury then upregulated by 1.5- to 3.8-fold for the remainder of the experiment. Wounding’s greatest effect on organic acid and TCA cycle metabolism involved the upregulation of an NADP^+^-dependent malic enzyme gene. NADP-ME catalyzes the decarboxylation of malate, the penultimate metabolite in the TCA cycle, to pyruvate and CO_2_, with a reduction of NADP^+^ to NADPH. This reaction serves to deplete the concentrations of a TCA cycle carbon intermediate, increase the concentration of the end-product of glycolysis, and increase the ratio of NADPH to NADP^+^, an indicator of cytoplasmic reducing potential. Increased NADP-ME expression in wounded tissues has been reported previously for several plant species, where it is speculated to promote cell wall strengthening and phytoalexin biosynthesis by increasing the availability of NADPH utilized in these processes^48,49^.

### Wounding alters carbon metabolism

The observed changes in transcriptional and enzymatic activities of sucrolytic reactions, glycolysis, the pentose phosphate pathway, the TCA cycle, and fermentation indicate that sugarbeet roots undergo significant changes in primary carbon metabolism in response to wounding. This is perhaps not surprising since wounding induces an upsurge in biosynthetic reactions, including those responsible for the synthesis of compounds used to seal off wound sites, such as callose, suberin, lignin, and their carbohydrate and phenolic precursors, and increases the synthesis of compounds that limit pathogen infection, such as defense-related proteins and phytoalexins^50,51^. This upsurge in biosynthesis requires carbon substrates which are largely obtained by diverting intermediates from primary carbon metabolism into secondary metabolic pathways. Glycolysis is a particularly important resource for wound-induced biosynthesis, since intermediates and products of this pathway are substrates for the synthesis of polyphenolic compounds, amino acids, alkaloids, and cell walls^25,32,52^.

It is perhaps not surprising, then, that glycolytic gene expression and glycolytic enzyme activities, especially those with a role in the regulation of flux through the pathway, were significantly upregulated in wounded sugarbeet roots. Wound induction of glycolytic gene expression has similarly been reported in other plant species and organs^53,54^. Although sucrolytic enzymes that generate substrates for glycolysis were largely unaffected by wounding, sugarbeet roots at harvest contained abundant quantities of the glycolytic substrates, glucose and fructose, and these compounds could be generated without altering sucrolytic transcripts or translated products since the in vivo activity of sucrose synthase, the predominant sucrolytic activity in harvested roots, is abundantly present in roots and can increase in response to alterations in cytoplasmic pH and reaction product concentration^55,56^. Nevertheless, utilization of glycolytic substrates clearly exceeded their generation as evidenced by the approximately 50% reduction in glucose concentration in injured roots.

Glycolytic induction was not accompanied by TCA cycle upregulation, which presumably necessitated the activation of fermentation to maintain cell metabolism in injured sugarbeet roots. TCA cycle capacity was at best unaffected, but more likely was reduced by injury since wounding downregulated or transiently downregulated two TCA cycle genes and upregulated a NADP^+^-ME gene that recycles a TCA cycle intermediate back to glycolysis. Since fermentation was highly upregulated in wounded roots, it seems probable that the TCA cycle and downstream pathways were insufficiently able to recycle glycolytically-produced NADH to NAD^+^, which would cause glycolysis to slow or cease and reduce carbon substrate availability for secondary metabolic pathways. Fermentation, however, provides a bypass of the TCA cycle by metabolizing the end-products of glycolysis, i.e., NADH and pyruvate, to allow continued operation of the glycolytic pathway^19^. In other plant species, fermentation is upregulated by ethylene^57^. It is notable, therefore, that the expression of genes involved in ethylene biosynthesis and signal transduction are highly upregulated in wounded sugarbeet roots^14^.

## Conclusion

Wound-induced changes in the gene expression and activities of enzymes in sugarbeet root primary metabolic pathways indicate that the increased demand for carbon substrates for the repair and defense of wounded tissues is met by a rapid and intense upregulation of glycolysis and fermentation, with minimal change in sucrolytic enzyme activities or the TCA cycle. That glycolysis was upregulated in response to injury is consistent with previous studies that found that this pathway is likely responsible for regulating and restricting primary carbon metabolism in postharvest sugarbeet roots. Additionally, since sucrolytic activities were generally unaffected by wounding, the abundant activity of sucrose synthase was presumably sufficient to provide the needed substrates for glycolysis, while the unresponsiveness of the TCA cycle and intense upregulation of fermentation indicate an inability of the TCA cycle to metabolize glycolytic end products that necessitated the engagement of fermentation for continued operation of glycolysis. That wounding induces fermentation should concern the sugarbeet industry since wounds are ubiquitous on stored roots and fermentation requires greater catabolism of sucrose to generate equivalent quantities of metabolic energy in the form of ATP and inefficiently utilizes the carbon released by sucrose catabolism for downstream biosynthetic reactions relative to the TCA cycle. Overall, this research establishes a central role for glycolysis and fermentation in generating metabolic substrates for the repair and defense of sugarbeet taproots injured during harvest and piling. While the induction of glycolysis was expected based on current knowledge of carbohydrate metabolism in postharvest sugarbeet roots, the induction of fermentation was both surprising and concerning since inefficiencies associated with this metabolic pathway are counterproductive to the maintenance of sucrose content during storage.

### Material and methods

#### Plant material and treatments

Forty-eight sugarbeet taproots of variety VDH66156 (SESVanderHave, Tienen, Belgium) were produced in a greenhouse in 15 L pots under a 16 h day/8 h night regimen, as previously described^24^. Taproots were removed from pots 16 weeks after planting. All leaf and petiole material were removed from the roots with a sharp knife, and roots were randomly divided into two groups of 24. One group of roots was gently hand washed, air dried, and used as controls; the remaining roots were washed and wounded by tumbling in a pilot-scale beet washer for 15 minutes^58^. Fifteen minutes (0.25 h) after administering the wound treatment, tissue samples were collected from four control and four wounded taproots by excising a transverse section from the middle portion of the taproot that included periderm and all underlying tissues up to the root central vascular cylinder. Samples were rapidly frozen in liquid nitrogen, lyophilized, and ground to a fine powder. The remaining roots of both treatments were incubated at 25 °C, with four roots from each treatment removed and sampled as described after 2, 4, 8, 12, and 24 h post-wounding. Individual taproots served as experimental replicates, with four replicates per time point x wounding treatment combination. All tissue samples were stored at −80 °C prior to analysis.

#### RNA sequencing

Total RNA was extracted and sequenced as previously described^14^ using a DNBseq platform (BGI Americas, Cambridge, MA, USA). Greater than 45 M raw reads and > 40 M clean reads were generated from each sample. Reads were mapped to the sugarbeet genome^59^ and analyzed for gene expression, differential gene expression, and biological function as previously described^14^.

#### Enzyme activity assays

Soluble proteins were extracted from lyophilized tissue and enzyme activities were quantified using established protocols. Proteins were extracted using eight volumes (w/v) of extraction buffer for all enzyme determinations except for pyruvate kinase which was extracted using ten volumes (w/v), with all extraction operations performed at 4 °C. Composition of extraction buffers was tailored to the activity being measured and are described in the references provided below for each enzyme activity. All enzyme activities were quantified using duplicate assays, such that all activities are the average of four biological and two technical replicates. All activities are expressed as a function of total soluble protein, with total protein concentration determined using Bio-Rad Protein Assay Dye Reagent (Hercules, CA, USA) according to the manufacturer’s protocol with bovine serum albumin used as the protein standard. Activities of the sucrolytic enzymes, sucrose synthase and soluble acid invertase, were determined using spectrophotometric end-point assays as described^30^, with standard curves generated using varying concentrations of fructose. Activities of the glycolytic enzymes, hexokinase (HXK) and phosphofructokinase (PFK), were assayed using the protocols of Moorhead and Plaxton^60^; glyceraldehyde-3-phosphate dehydrogenase (GAPDH) activity was assayed using the protocol of Burrel et al.^61^, and pyruvate kinase (PK) activity was quantified using a pyruvate kinase assay kit from Sigma-Aldrich (St. Louis, MO, USA) according to the kit’s protocol. Activities of the ethanol biosynthetic pathway enzymes, pyruvate decarboxylase (PDC) and alcohol dehydrogenase (ADH) were determined using the methods of Chang et al.^62^ and Chung and Ferl^63^, respectively. Rates of NADH oxidation or NAD^+^ reduction for PDC and ADH assays, respectively, were measured at 15 s intervals for 3 min at 25 °C.

#### Carbohydrate quantifications

Soluble carbohydrates were extracted from lyophilized tissue using 80% methanol and analyzed for sucrose, glucose, and fructose concentrations by high performance anion exchange chromatography and electrochemical detection as previously described^64^.

#### Data analysis

Heat maps were constructed in Origin2021b (OriginLab Corp., Northampton, MA, USA) using the log_2_ fold change in fragments per kilobase of transcript per million mapped reads (FPKM) between wounded and unwounded control roots at each time point. Significant differences in enzyme activities or carbohydrate concentrations between control and wounded roots for a time point were identified using t-tests in Microsoft Excel 365 (Redmond, WA, USA). The data was subjected to analysis of variance (ANOVA) to identify treatment differences in enzyme activities and carbohydrate concentrations using MiniTab Statistical software, ver. 16.0 (State College, PA, USA). For all statistical analyses, *p* ≤ 0.05.

## Supplementary Information

Supplementary Information.

## Data Availability

Sequence data that support the findings of this study have been deposited in the National Center for Biotechnology Information (NCBI) Sequence Read Archive (SRA) at http://www.ncbi.nlm.nih.gov/, under BioProject number PRJNA762331.
